# Spending Changes After Moving to Areas With Greater ACO Participation Among Nonattributed Medicare Beneficiaries

**DOI:** 10.1001/jamanetworkopen.2024.58311

**Published:** 2025-02-20

**Authors:** Yucheng Hou, Marisa Elena Domino, Valerie A. Lewis, Qing Gong, Kevin Callison, Justin G. Trogdon

**Affiliations:** 1 Department of Management, Policy and Community Health, School of Public Health, The University of Texas Health Science Center at Houston, Houston; 2 Center for Health Information and Research, College of Health Solutions, Arizona State University, Phoenix; 3 Gillings School of Global Public Health, The University of North Carolina at Chapel Hill, Chapel Hill; 4 Department of Economics, The University of North Carolina at Chapel Hill, Chapel Hill; 5 Department of Health Policy and Management, Celia Scott Weatherhead School of Public Health and Tropical Medicine, Murphy Institute for Political Economy, Tulane University, New Orleans, Louisiana

## Abstract

**Importance:**

Accountable care organizations (ACOs) under the Medicare Shared Savings Program have long been envisioned as a pathway to improved care efficiency and quality of care for all Medicare beneficiaries. However, little is known about whether the changes in health care spending associated with ACOs have extended beyond ACO-attributed Medicare beneficiaries to all Medicare beneficiaries.

**Objective:**

To estimate spending changes by non-ACO–attributed Medicare beneficiaries after moving to geographic areas with greater ACO participation.

**Design, Setting, and Participants:**

This repeated cross-sectional study analyzed claims from a 20% representative sample of all Medicare beneficiaries, aged 65 to 99 years, from 2009 to 2017. The sample consisted of Medicare beneficiaries who were never attributed to an ACO and moved once across hospital service areas (HSAs) during the study period (movers) and was supplemented by a 20% random sample of beneficiaries who never moved (nonmovers). Data analysis took place from November 2022 to October 2024.

**Exposure:**

Changes in the ACO market penetration rate triggered by nonattributed Medicare beneficiaries moving across HSAs.

**Main Outcomes and Measures:**

Annual standardized Medicare spending per beneficiary on acute inpatient, outpatient facility, physician services, and total acute care as well as on hospital outpatient department, evaluation and management, and nonadmitted emergency department visits.

**Results:**

The estimation sample included 62 618 movers (388 263 beneficiary-years; mean [SD] age, 75 [7] years; 134 503 [65%] female-years) and 433 298 nonmovers (2 066 404 beneficiary-years; mean [SD] age, 76 [8] years; 1 273 154 [62%] female-years). In the base model, moving into a market with a 1-SD higher ACO penetration was associated with a 5.8% (95% CI, 4.1%-7.4%) decrease in spending on outpatient facilities and with a 1.6% (95% CI, 0.9% to 2.2%) increase in spending on physician services. Limited changes in total spending on acute inpatient and total acute care were found. These estimated changes were robust to controlling for a rich set of patient- and market-level characteristics and origin-destination HSA combinations.

**Conclusions and Relevance:**

This study provided novel evidence of market-level ACO spillovers to non-ACO–attributed Medicare beneficiaries. Although no substantial ACO spillovers in total acute care spending occurred, the substitution in spending on outpatient facility and physician services suggested that outpatient care may shift away from higher-cost facility settings for all Medicare beneficiaries in markets with greater ACO penetration.

## Key Points

**Question:**

Are there changes in health care spending by non–accountable care organization (ACO)–attributed Medicare beneficiaries after a move to geographic areas with greater ACO participation?

**Findings:**

In this repeated cross-sectional study of 62 618 mover and 433 298 nonmover Medicare beneficiaries, non-ACO–attributed beneficiaries’ move to areas with more Medicare beneficiaries in ACOs was associated with reduced outpatient facility spending and increased physician services spending. The changes in spending on acute inpatient or total acute care were minimal.

**Meaning:**

These findings suggest that although no substantial spillovers from ACOs to nonattributed beneficiaries occurred, outpatient care may shift away from higher-cost facility settings in markets with greater ACO penetration.

## Teaser

This cross-sectional study examines health care use and spending patterns associated with the relocation of Medicare beneficiaries to areas with high accountable care organization (ACO) penetration rates.

## Introduction

Over the past decade, health care delivery in the US has been transformed by the rapid expansion of alternative payment models, shifting the focus from fee-for-service to value-based care. The Medicare Shared Savings Program (MSSP), one of the largest alternative payment models, is a form of accountable care organization (ACO) wherein a network of physician groups, hospitals, and other facility participants collectively assume responsibility for spending and quality for an attributed traditional Medicare (hereafter Medicare) population in exchange for shared savings or losses. As of 2024, the MSSP has reached approximately 11 million Medicare beneficiaries,^1^ with the Centers for Medicare & Medicaid Services (CMS) aiming to align all remaining Medicare beneficiaries with an accountable care relationship by 2030.^2^

To date, researchers and policymakers have predominately examined the impact of ACOs among attributed beneficiaries. Modest savings, which appeared to be concentrated among physician-led ACOs, have been well documented and can be attributed to reductions in spending on acute inpatient care, outpatient care in hospital-owned facilities, and postacute care.^3,4,5,6,7,8^ Yet, with the promise of expanding ACO models to all Medicare beneficiaries, it is important to understand the overall impact of the MSSP, which may extend beyond the attributed Medicare beneficiaries and may be associated with either greater or smaller savings than currently estimated.^9,10^ Previous studies have focused on ACO spillovers (ie, spread to nontargeted population) within organizations or across payers, including spillovers in postacute care in participating hospitals,^11^ in commercial spending,^12^ and to Medicare Advantage.^13^ However, whether ACOs are associated with changes in outcomes for nonattributed Medicare beneficiaries at the market level remains unknown.

There are several spillover channels through which the impact of ACOs may extend to nonattributed Medicare beneficiaries. First, spillovers may occur due to the same participating practices treating both attributed and nonattributed beneficiaries. Before 2017, beneficiaries were retrospectively attributed to an ACO based on where they received the plurality of their primary care services. Participating practices could care for beneficiaries attributed to their ACO based on the plurality rule, beneficiaries attributed to another ACO, and nonattributed beneficiaries at the same time. Hence, the clinical transformation and infrastructure investment incentivized by ACO participation may affect the practice’s entire patient panel, potentially resulting in spillovers that align with the changes observed among attributed patients.^14,15,16^ Alternatively, caring for both attributed and nonattributed patients may be associated with different incentives and practice patterns by attribution status, leading to spillovers in the opposite direction.^14,15,16^ Second, spillovers may also occur through learning or partnership across practices.^9,17^ Physician-led ACOs may collaborate with non-ACO local hospitals to coordinate care across settings, which may also bring changes to non-ACO practices that align with those observed in participating practices.

Ideally, studying ACO spillovers would involve randomly assigning some areas with ACOs and others without ACOs and then comparing outcomes for nonattributed beneficiaries.^16^ However, it is challenging to study spillover in reality because the adoption of ACOs is not random across places; thus, any analysis may run the risk of finding an outcome attributed to ACO spillover that is actually merely a function of which places are forming ACOs and which are not.^9,18^ To overcome this challenge, we used an alternative method: we studied nonattributed beneficiaries who moved from places with few ACOs to places with many ACOs, and vice versa. Given that the direction of the move was likely random, this approach allowed us to separate the role of ACO penetration from the role of other local market characteristics of areas where ACOs form. Similar designs have been developed to examine geographic variation in Medicare use pattern and care fragmentation across regions.^19,20^ We built on this foundational work to provide new evidence on ACO spillover in the context of ongoing Medicare payment reform. Specifically, we aimed to estimate spending changes by non-ACO–attributed Medicare beneficiaries after moving to geographic areas with greater ACO participation.

## Methods

The University of North Carolina at Chapel Hill Institutional Review Board approved this repeated cross-sectional study and waived the informed consent requirement because the risk involved in this research was no more than minimal. We followed the Strengthening the Reporting of Observational Studies in Epidemiology (STROBE) reporting guideline.^21^

### Study Setting and Sample

We used a 20% representative sample of all Medicare beneficiaries from 2009 to 2017. We included beneficiaries aged 65 to 99 years and who were eligible for attribution to an MSSP ACO by being continuously enrolled in Medicare Parts A and B, not being enrolled in Medicare Advantage, and having at least 1 eligible evaluation and management service provided by a primary care physician each year. Beneficiaries were attributed to an ACO using the MSSP provider (ie, practices and practitioners)–level file based on CMS methods and previously outlined modifications^4,8^; beneficiaries attributed to the Pioneer ACO model were excluded from the analysis due to ACO dropouts before 2015.^4^

The sample for this study consisted of beneficiaries who were never attributed to an ACO during the study period, including those who moved exactly once across hospital service areas (HSAs) between 2012 and 2017 as well as a 20% random sample of nonmovers (eFigure 1 in Supplement 1). We used 3436 HSAs, defined by the Dartmouth Atlas of Health Care based on travel patterns to local hospitals,^22^ to characterize local health care markets.^23^ Eligible beneficiaries were identified as movers if their HSA of residence, assigned based on the zip code recorded in the Master Beneficiary Summary File (MBSF), changed exactly once between 2012 and 2017. Since the residence in the MBSF was finalized at the end of each calendar year, a different HSA from the previous year suggested that the beneficiary moved at some point during the current year. The nonmovers were identified if their HSA of residence remained the same during the study period. We excluded beneficiaries who changed their HSAs of residence more than once during the study period, as they may have different health care use patterns.^17^ Similarly, to verify beneficiaries’ relocation, we excluded beneficiaries with fewer than 75% of their total acute care claims in their corresponding HSAs each year except the year of the move.^18^ The share of claims billed in destination HSAs before and after the move is illustrated in eFigure 2 in Supplement 1. We also excluded beneficiaries who moved prior to the onset of the MSSP in 2012 or in the last year of the study period in 2017 or who had missing values for any of the covariates (eMethods in Supplement 1).

### Exposure

The key exposure was the interaction between the change in ACO penetration triggered by beneficiaries moving across HSAs and an indicator of postmove years. We defined the ACO penetration rate as the share of eligible beneficiaries attributed to an ACO within an HSA year from 2012 to 2017. The destination-origin difference was calculated by subtracting premove ACO penetration in origin HSAs from postmove ACO penetration in destination HSAs; premove and postmove ACO penetration rates were computed as the mean ACO market penetration during the respective periods. eFigure 3 in Supplement 1 shows the distribution of changes in ACO penetration triggered by moving. The change in ACO penetration was normalized to 0 for nonmovers. We standardized the change using the mean (SD) of ACO penetration across all HSAs.

### Outcome Measures

The primary outcomes were the annual standardized Medicare spending on acute inpatient, outpatient facility, physician services, and total acute care per beneficiary as well as spending by point of service, including hospital outpatient department, evaluation and management, and nonadmitted emergency department (ED). We followed the CMS spending standardization approach to remove differences in the spending components due to geographic variation, such as wage index, indirect costs of medical education, and payments to disproportionate share hospitals.^24^ Secondary outcomes included annual counts of acute hospitalization, outpatient facility visits, hospital outpatient department visits, physician visits, evaluation and management visits, and nonadmitted ED visits per beneficiary. All outcomes were censored at the 99th percentile and log-transformed (plus a constant of 1) to address the skewed distribution. Details on these outcomes are provided in the eMethods in Supplement 1.

### Covariates

We controlled for time-varying patient- and market-level characteristics that may be associated with the timing of a move, with ACO penetration in origin and destination markets, and with outcomes. Patient-level characteristics included 27 chronic conditions from the Chronic Conditions Data Warehouse grouped into 10 categories (eTable 1 in Supplement 1). Race and ethnicity were self-reported by beneficiaries during Medicare enrollment and included Hispanic, non-Hispanic Black (hereafter Black), non-Hispanic White (hereafter White), and other (including American Indian or Alaska Native and Asian or Pacific Islander) categories. Race and ethnicity data were collected because previous studies have suggested that movers are more likely to be White individuals compared with nonmovers.^19^ Race and ethnicity designations were based on the Research Triangle Institute algorithm.^25^

Market-level characteristics included Medicare Advantage penetration (a proxy for experience in payment reform and risk contracts) and percentage of dually eligible beneficiaries (a proxy for patient risks) at the hospital referral region (HRR) level. We also included percentage of high school graduates, residents with a bachelor’s degree, and residents living below the federal poverty line at the zip code tabulation area level. We did not adjust for hierarchical condition category (HCC) scores in the main analysis because they are likely affected by ACO penetration; nonetheless, we tested the robustness of the results to the inclusion of these HCC risk scores as covariates.

### Statistical Analysis

We used a difference-in-differences framework to compare outcomes across beneficiaries with varying degrees of changes in ACO penetration (continuous treatment) triggered by movement across markets, before and after the move. We regressed each outcome on the interaction between changes in ACO penetration and an indicator for being in the postmove period. All regressions controlled for beneficiary fixed effects, calendar year fixed effects, and year relative to move fixed effects to account for time-invariant beneficiary characteristics, calendar year trends in outcomes, and year relative to the move trends in outcomes (up to 4 years before the move and 2 years after the move; 0 indicating year of the move), respectively. The year relative to the move corresponded to different calendar years for movers who moved at different periods and was normalized to 0 for nonmovers. We adjusted only for beneficiary age (categorized into 5-year bins) in the base model and sequentially included time-varying patient- and market-level characteristics to assess the robustness of the findings.

We conducted additional analyses to explore alternative explanations for the findings. First, we estimated an event study specification to examine whether the changes in outcomes had already begun before the move (pretrends), in which case the changes in outcomes may be associated with patients’ preferences on when and where to move (possibly in response to disease onset) instead of an increase or a decrease in ACO penetration. To minimize the impact of pretrends, we detrended the outcome variables using the estimated coefficients on a linear trend among movers in the premove period.^26,27,28^ Second, we assessed the extent to which the remaining differences between origin and destination HSAs other than ACO penetration were associated with the changes in outcomes. To minimize the impact of other uncontrolled differences between origin and destination markets, we compared spending changes among beneficiaries who moved from the same origin HSAs to the same destination HSAs at different times and, therefore, experienced different ACO penetration. We did so by replacing the beneficiary-level fixed effects with the combination origin-destination HSA fixed effects. Details on empirical methods are provided in the eMethods in Supplement 1.

In sensitivity analyses, we tested the robustness of the findings to an alternative market definition based on HRR, adjusted for HCC scores, and analyzed the outcomes without detrending and without log transformation. For all analyses, we computed 95% CIs based on SEs clustered at the beneficiary level. A 2-sided statistical significance threshold was set at *P* < .05. Data curation and statistical analyses were performed between November 2022 and October 2024 using SAS, version 9.4 (SAS Institute Inc) and Stata, version 17.0 (StataCorp LLC).

## Results

We included 62 618 movers (388 263 beneficiary-years) and 433 298 nonmovers (2 066 404 beneficiary-years) in the estimation sample. The Table compares baseline characteristics between nonmovers and movers before the move. Movers had a mean (SD) age of 75 (7) years and included 134 503 (65%) female-years and 73 097 (35%) male-years. Nonmovers had a mean (SD) age of 76 (8) years and included 1 273 154 (62%) female-years and 793 250 (38%) male-years. Compared with nonmovers, movers were less likely to be dually eligible for Medicaid (17% vs 21%; standardized difference, 0.10) and to be healthier, as evidenced by lower mean (SD) HCC scores (1.1 [0.9] vs 1.3 [1.1]; standardized difference, 0.18) and fewer mean (SD) number of chronic conditions (3.4 [2.4] vs 3.8 [2.6]; standardized difference, 0.15). Overall, movers and nonmovers had comparable annual mean (SD) total acute care spending at baseline ($7947 [$11 457] vs $8364 [$12 457]; standardized difference, 0.03).

**Table.** Baseline Characteristics of Nonattributed Beneficiary-Years by Moving Status^a^

	Beneficiary-years, mean (SD)		Standardized difference^b^
	Nonmovers (n = 2 066 404)	Movers (n = 207 600)	
**Demographic and clinical characteristics**			
Age, y	76 (8)	75 (7)	0.15
Sex, No. (%)			
Female	1 273 154 (62)	134 503 (65)	0.07
Male	793 250 (38)	73 097 (35)	0.07
Race and ethnicity, No. (%)^c^			
Hispanic	42 518 (2)	3154 (2)	0.04
Non-Hispanic Black	158 048 (8)	9068 (4)	0.14
Non-Hispanic White	1 752 750 (85)	185 457 (89)	0.13
Other^d^	98 151 (5)	8907 (4)	0.02
Dually eligible for Medicaid, No. (%)	434 819 (21)	35 705 (17)	0.10
Disability, No. (%)	15 772 (1)	2662 (1)	0.05
ESKD, No. (%)	9515 (0.5)	519 (0.3)	0.04
HCC score	1.3 (1.1)	1.1 (0.9)	0.18
No. of chronic conditions	3.8 (2.6)	3.4 (2.4)	0.15
**Standardized spending, $**			
Acute inpatient	2418 (6609)	2108 (6114)	0.05
Outpatient facility	1534 (3047)	1472 (2850)	0.02
Hospital outpatient department	1088 (2209)	1056 (2121)	0.01
Physician services	3734 (4172)	3867 (4159)	0.03
Evaluation and management	914 (655)	943 (660)	0.04
Nonadmitted ED	298 (663)	276 (633)	0.03
Total acute care	8364 (12 457)	7947 (11 457)	0.03
**Service use counts**			
Acute hospitalization	0.2 (0.6)	0.2 (0.5)	0.05
Outpatient facility visits	4.8 (5.8)	4.6 (5.5)	0.03
Hospital outpatient department visits	3.0 (4.1)	2.9 (3.9)	0.03
Physician visits	23.7 (19.6)	23.6 (18.8)	0.01
Evaluation-and-management visits	8.4 (6.0)	8.6 (6.0)	0.04
Nonadmitted ED visits	0.6 (1.1)	0.5 (1.0)	0.05

Abbreviations: ED, emergency department; ESKD, end-stage kidney disease; HCC, hierarchical condition category.

^a^ Baseline characteristics were based on the premove periods for moving beneficiaries and all time periods for nonmoving beneficiaries.

^b^ A less than 0.10 standardized difference in means indicates a negligible difference between movers and nonmovers.

^c^ Race and ethnicity were self-reported by beneficiaries during the Medicare enrollment and designations were enhanced by the Research Triangle Institute algorithm.^25^

^d^ Other included American Indian or Alaska Native and Asian or Pacific Islander because of the small sample sizes for these groups.

**Figure 1.**
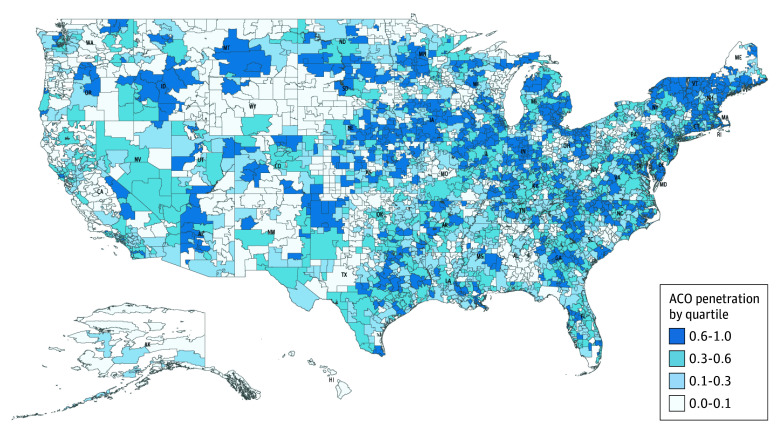
Medicare Shared Savings Program (MSSP) Accountable Care Organization (ACO) Penetration Rates by Hospital Service Area (HSA) Penetration rate was calculated as the share of all eligible beneficiaries, derived from a 20% Medicare sample, attributed to an MSSP ACO in an HSA in 2017.

Figure 1 shows the geographic distribution of ACO penetration in 2017 across HSAs by quartiles. HSAs with ACO penetration in the highest quartile were located along the coast and in urban areas, while more HSAs in the lowest ACO penetration quartile were in the West region. eFigure 4 in Supplement 1 shows ACO penetration at both the HSA and HRR levels, with greater variation at the HSA (local health care market) level compared with the HRR level. On average, movers tended to move from low to high ACO penetration HSAs, with an 18–percentage point increase in ACO penetration (eFigure 3 in Supplement 1).

**Figure 2.**
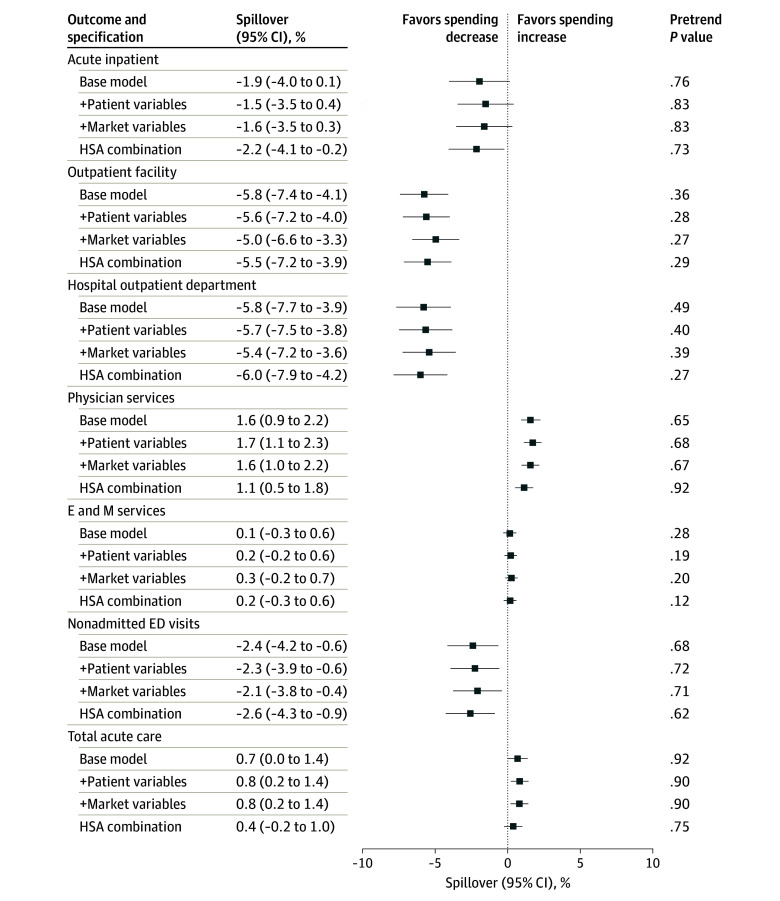
Estimated Spending Changes After Moving to Areas With 1-SD Higher Share of Medicare Beneficiaries in Accountable Care Organizations for Nonattributed Beneficiaries All regressions were controlled for age (5-year age bins); beneficiary (specification 1-3); or fixed effects for hospital service area (HSA) combination (specification 4), year relative to move, and calendar year. Pretrend tests reported the *P* value from a joint *F* test of the estimated coefficients during the premove period after the detrending adjustment. *P* < .05 indicates evidence of pretrend. Error bars represent 95% CIs, and the + symbol indicates that the variables were sequentially added to the base model. E and M indicates evaluation and management; ED, emergency department.

**Figure 3.**
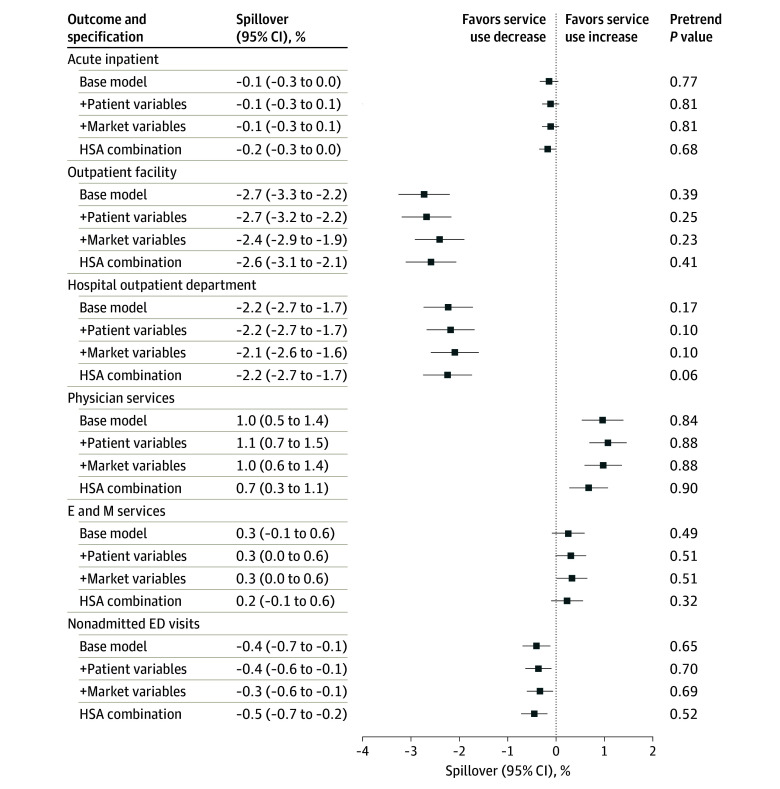
Estimated Service Use Changes After Moving to Areas With 1-SD Higher Share of Medicare Beneficiaries in Accountable Care Organizations for Nonattributed Beneficiaries All regressions were controlled for age (5-year age bins); beneficiary (specification 1-3); or fixed effects for hospital service area (HSA) combination (specification 4), year relative to move, and calendar year. Pretrend tests reported the *P* value from a joint *F* test of the estimated coefficients during the premove period after the detrending adjustment. *P* < .05 indicates evidence of pretrend. Error bars represent 95% CIs, and the + symbol indicates that the variables were sequentially added to the base model. E and M indicates evaluation and management; ED, emergency department.

Figure 2 plots the estimated changes in spending after moving to areas with higher ACO penetration for nonattributed beneficiaries by different specifications. In the base model, moving into a market with a 1-SD higher ACO penetration was associated with a 5.8% (95% CI, 4.1%-7.4%) decrease in spending on outpatient facility and a 1.6% (95% CI, 0.9% to 2.2%) increase in spending on physician services. While we observed a 1.9% (95% CI, −4.0% to 0.1%) decrease in spending on acute inpatient hospitalization, the change was not statistically significant. Together, these changes translated into an increase of 0.7% (95% CI, 0% to 1.4%) in total acute care spending. When examining spending changes by point of service, we also found reductions in spending on hospital outpatient department visits (−5.8%; 95% CI, −7.7% to −3.9%) and nonadmitted ED visits (−2.4%; 95% CI, −4.2% to −0.6%). These results were largely robust to controlling for patient-level characteristics, market-level characteristics, and HSA-combination fixed effects. When examining service use (Figure 3), we observed similar decreases in outpatient facility visits (−2.7%; 95% CI, −3.3% to −2.2%), hospital outpatient department visits (−2.2%; 95% CI, −2.7% to −1.7%), and nonadmitted ED visits (−0.4%; 95% CI, −0.7% to −0.1%) and increases in physician visits (1.0%; 95% CI, 0.5% to 1.4%) associated with a move.

In sensitivity analyses, we found that using a larger market definition (ie, HRRs) produced qualitatively similar results, although reduced spending on outpatient facilities was not significant (–0.8%; 95% CI, –3.2% to 1.5%) (eFigures 5 and 6 in Supplement 1). All results were robust to controlling for HCC scores (eTable 2 in Supplement 1). Although we observed evidence of downward pretrends in spending on outpatient facility visits and hospital outpatient department visits, detrending adjustments did not change the direction or significance of the findings (eTable 3, eFigures 7 and 8 in Supplement 1). We also found similar results when using outcomes that had not been log-transformed due to adjustment for high frequency of 0s in certain point-of-service outcomes (ie, acute hospitalization) (eTable 4 in Supplement 1). Changes in outpatient facility and physician services were consistently robust, while changes in acute hospitalization, evaluation and management, and total acute care were more sensitive to alternative specifications.

## Discussion

In this repeated cross-sectional study, we provided novel evidence of ACO spillovers to nonattributed beneficiaries by examining their spending and service use change after moving to areas with higher ACO penetration. Overall, we found limited evidence of ACO spillovers in acute inpatient or total acute care spending. However, when assessing spillovers by different types of service and care settings, we found consistent evidence of an offset in outpatient care use patterns by care settings. Moving into a market with higher ACO penetration was associated with reduced spending on outpatient facilities and increased spending on physician services. Reductions in spending on hospital outpatient department visits and nonadmitted ED visits also indicate a shift away from higher-cost facility settings.

These findings add to the existing evidence of the lack of ACO spillovers on postacute care use for all Medicare beneficiaries^11^ and the privately insured population.^12^ The lack of spillovers is consistent with the limited savings associated with ACOs for attributed beneficiaries, which makes it unlikely for substantial spillovers to occur for nonattributed beneficiaries. The incentives for learning and collaboration across practices (external to ACOs) may be even weaker. The offset between spending on outpatient facilities and spending on physician services was aligned with the existing evidence of ACOs on attributed beneficiaries: a similar substitution was observed in outpatient care spending for independent physician offices vs hospital-owned facilities among physician-led ACOs.^4,8^ The shift away from higher-cost facility settings to lower-cost practices is central to payment reform and aligns with ACOs' incentive to reduce spending through better care coordination, preventive care services, and chronic care management.^29,30^ Given that most of the savings on attributed beneficiaries were generated by physician-led ACOs rather than hospital-led ACOs, future work is needed to explore heterogeneity in spillovers by ACO types, both within Medicare and to other payers.

The findings of this study can be better contextualized with the well-documented spillover of Medicare Advantage plans. The increase in Medicare Advantage penetration has been associated with reduced hospital costs, use, and treatment intensity for traditional Medicare beneficiaries.^14,15,31,32,33^ Although ACOs and Medicare Advantage plans likely provide similar channels through which systemwide spillovers can occur,^14,15^ they have distinct designs, implying differences in spillover magnitudes. For example, capitated payments under Medicare Advantage plans tend to have greater financial incentives for contracted practices and practitioners to reduce spending. The financial incentives under ACOs, however, may be weaker due to 1-sided risk and retrospective attribution (ACOs moved toward 2-sided risks and prospective attribution under the Pathways to Success program after 2019). Additionally, ACOs allow beneficiaries flexibility in choosing practices and practitioners, unlike Medicare Advantage plans, which have practice and practitioner networks and utilization management tools. Without these incentives, the ACO spillover may be smaller in magnitude.

### Limitations

This study has several limitations. First, although we controlled for market-level Medicare Advantage penetration as an overall proxy for market experience with risk-based contracts,^23^ the change in ACO penetration may coincide with other payment reforms. Similarly, non-ACO–attributed Medicare beneficiaries may be attributed to other payment models, such as bundled payments, which we could not observe in our data. Second, we observed downward pretrends in spending on and use of outpatient facilities, which may indicate patient preferences in choosing the destination of their move, although the offset between outpatient facility and physician services was still robust after detrending adjustment. Third, the zip code recorded in the MBSF may not necessarily reflect residency as it is the mailing address for official correspondence from the Social Security Administration. We limited the analysis to beneficiaries who had at least 75% of the total claims each year (except the year of move) in their corresponding HSAs to further verify the mover status.

## Conclusions

In this repeated cross-sectional study, we provided novel evidence of market-level ACO spillovers to non-ACO–attributed Medicare beneficiaries. Although we did not find substantial spillovers in total acute care spending from ACOs to nonattributed Medicare beneficiaries, the substitution in spending on outpatient facility and physician services suggested that outpatient care may shift away from higher-cost facility settings for all Medicare beneficiaries in markets with greater ACO penetration.

## Notes

Supplement 1.
eMethods.
**eTable 1.** Chronic Condition Categories **eTable 2.** Sensitivity Analysis: Estimated Changes After Moving to HSAs With More Medicare Beneficiaries in Accountable Care Organizations for Non-attributed Beneficiaries Using Alternative Specifications (Adjusting for Hierarchical Condition Categories) **eTable 3.** Sensitivity Analysis: Estimated Changes After Moving to HSAs With More Medicare Beneficiaries in Accountable Care Organizations for Non-attributed Beneficiaries Using Alternative Specifications (Without Detrending) **eTable 4.** Sensitivity Analysis: Estimated Changes After Moving to HSAs With More Medicare Beneficiaries in Accountable Care Organizations for Non-attributed Beneficiaries Using Alternative Specifications (Outcomes without Log Transformation) **eFigure 1.** Diagram of Study Sample Selection **eFigure 2.** Share of Claims Billed in Destination Areas by Year Relative to Move **eFigure 3.** Distribution of Changes in the HSA-level ACO Penetration Induced by Movers **eFigure 4.** Variation in Accountable Care Organization Penetration at the HSA and HRR Levels **eFigure 5.** Sensitivity Analysis: Estimated Spending Changes After Moving to HRRs With More Medicare Beneficiaries in Accountable Care Organizations for Non-attributed Beneficiaries **eFigure 6.** Sensitivity Analysis: Estimated Service Use Changes After Moving to HRRs With More Medicare Beneficiaries in Accountable Care Organizations for Non-attributed Beneficiaries **eFigure 7.** Event Study Graph: Estimated Spending Changes After Moving to Hospital Service Areas [HSA] With More Medicare Beneficiaries in Accountable Care Organizations [ACO] for Non-attributed Beneficiaries **eFigure 8.** Event Study Graph: Estimated Service Use Changes After Moving to HSAs With More Medicare Beneficiaries in Accountable Care Organizations for Non-attributed Beneficiaries
eReferences

Supplement 2.
Data Sharing Statement
