# Supplementary material for: Spending Changes After Moving to Areas With Greater ACO Participation Among Nonattributed Medicare Beneficiaries
Source: JAMA Netw Open. 2025 Feb 20;8(2):e2458311. doi: 10.1001/jamanetworkopen.2024.58311 (PMC11843370; doi:10.1001/jamanetworkopen.2024.58311)
Supplement: Supplement 1. — eMethods. eTable 1. Chronic Condition Categories eTable 2. Sensitivity Analysis: Estimated Changes After Moving to HSAs With More Medicare Beneficiaries in Accountable Care Organizations for Non-attributed Beneficiaries Using Alternative Specifications (Adjusting for Hierarchical Condition Categories) eTable 3. Sensitivity Analysis: Estimated Changes After Moving to HSAs With More Medicare Beneficiaries in Accountable Care Organizations for Non-attributed Beneficiaries Using Alternative Specifications (Without Detrending) eTable 4. Sensitivity Analysis: Estimated Changes After Moving to HSAs With More Medicare Beneficiaries in Accountable Care Organizations for Non-attributed Beneficiaries Using Alternative Specifications (Outcomes without Log Transformation) eFigure 1. Diagram of Study Sample Selection eFigure 2. Share of Claims Billed in Destination Areas by Year Relative to Move eFigure 3. Distribution of Changes in the HSA-level ACO Penetration Induced by Movers eFigure 4. Variation in Accountable Care Organization Penetration at the HSA and HRR Levels eFigure 5. Sensitivity Analysis: Estimated Spending Changes After Moving to HRRs With More Medicare Beneficiaries in Accountable Care Organizations for Non-attributed Beneficiaries eFigure 6. Sensitivity Analysis: Estimated Service Use Changes After Moving to HRRs With More Medicare Beneficiaries in Accountable Care Organizations for Non-attributed Beneficiaries eFigure 7. Event Study Graph: Estimated Spending Changes After Moving to Hospital Service Areas [HSA] With More Medicare Beneficiaries in Accountable Care Organizations [ACO] for Non-attributed Beneficiaries eFigure 8. Event Study Graph: Estimated Service Use Changes After Moving to HSAs With More Medicare Beneficiaries in Accountable Care Organizations for Non-attributed Beneficiaries eReferences [file jamanetwopen-e2458311-s001.pdf]

Supplemental Online Content

Hou Y, Domino ME, Lewis VA, Gong Q, Callison K, Trogdon JG. Spending changes after moving to areas with greater accountable care organization participation among nonattributed Medicare beneficiaries. *JAMA Netw Open*. 2025;8(2):e2458311. doi:10.1001/jamanetworkopen.2024.58311

eMethods

eTable 1. Chronic Condition Categories

eTable 2. Sensitivity Analysis: Estimated Changes After Moving to HSAs With More Medicare Beneficiaries in Accountable Care Organizations for Non-attributed Beneficiaries Using Alternative Specifications (Adjusting for Hierarchical Condition Categories)

eTable 3. Sensitivity Analysis: Estimated Changes After Moving to HSAs With More Medicare Beneficiaries in Accountable Care Organizations for Non-attributed Beneficiaries Using Alternative Specifications (Without Detrending)

eTable 4. Sensitivity Analysis: Estimated Changes After Moving to HSAs With More Medicare Beneficiaries in Accountable Care Organizations for Non-attributed Beneficiaries Using Alternative Specifications (Outcomes without Log Transformation)

eFigure 1. Diagram of Study Sample Selection

eFigure 2. Share of Claims Billed in Destination Areas by Year Relative to Move

eFigure 3. Distribution of Changes in the HSA-level ACO Penetration Induced by Movers

eFigure 4. Variation in Accountable Care Organization Penetration at the HSA and HRR Levels

eFigure 5. Sensitivity Analysis: Estimated Spending Changes After Moving to HRRs With More Medicare Beneficiaries in Accountable Care Organizations for Non-attributed Beneficiaries

eFigure 6. Sensitivity Analysis: Estimated Service Use Changes After Moving to HRRs With More Medicare Beneficiaries in Accountable Care Organizations for Non-attributed Beneficiaries

eFigure 7. Event Study Graph: Estimated Spending Changes After Moving to Hospital Service Areas [HSA] With More Medicare Beneficiaries in Accountable Care Organizations [ACO] for Non-attributed Beneficiaries

eFigure 8. Event Study Graph: Estimated Service Use Changes After Moving to HSAs With More Medicare Beneficiaries in Accountable Care Organizations for Non-attributed Beneficiaries

eReferences

This supplemental material has been provided by the authors to give readers additional information about their work.

eMethods

1. Supplemental data source description

Research Identifiable Data (RIF)

Medicare Claims (20% Sample): We used a 20% sample of Medicare RIF to extract all Medicare beneficiaries aged 65 to 99 years and their medical claims, including inpatient, outpatient, and carrier from 2009 to 2017. Medicare Beneficiary Summary Files (MBSF) were used to identify Medicare enrollment in Parts A & B and Medicare Advantage plans as well as other beneficiary demographics.

MSSP ACO Provider Level RIF: We used the shared savings provider (ie, practices and practitioners)-level RIF from 2013 to 2017 to identify which Medicare-enrolled practices participated in MSSP ACOs; practices were identified by tax identification numbers (TINs) or Centers for Medicare & Medicaid Services (CMS) certification numbers (CCNs). Additionally, we used the Pioneer ACO provider file from 2012-2014 to exclude beneficiaries that were ever assigned to a Pioneer ACO.

Medicare Data on Physician Practice and Specialty (MD-PPAS): We used MD-PPAS RIF from 2011-2016 to extract a set of physicians and their specialties linked to the 20% claims data.

Area-level Characteristics

American Community Survey: The U.S. Census Bureau's American Community Survey (ACS) data is a nationwide survey that provides information on social, economic, housing, and demographic characteristics of the national population. We used the five-year ACS data to define beneficiary area-level poverty and educational attainment for each year of the study, including ZIP code tabulation area (ZCTA) poverty (share of residents living below 100% of the federal poverty level) and educational attainment (share of residents with high school diploma or equivalent and with bachelor's degree or higher). We linked beneficiaries to ZCTA-level ACS data each year using the beneficiary ZIP codes recorded in MBSF.

Medicare Geographic Variation Public Use File (PUF): The CMS created the geographic variation PUF for researchers and policymakers to evaluate health care utilization and spending trends by geography for the Medicare fee-for-service population. The file includes demographic, spending, utilization, and quality indicators

© 2025 Hou Y et al. *JAMA Network Open*

at the state, hospital referral region (HRR), and county levels. We used the geographic variation PUF to obtain area-level covariates, including the share of dually eligible beneficiaries, average Hierarchical Condition Category (HCC) scores, and the Medicare Advantage participation rate at the HRR level.

Federal regulation

Inpatient Prospective Payment System (IPPS) Final Rule: The CMS sets IPPS payments based on operating and capital costs (base payment amounts), Diagnosis Related Group (DRG) relative weights, and market condition adjustments (wage index). The tables and files used to prepare IPPS final rules are published on the CMS website annually. We used the base payment amounts, DRG relative weights, and wage index tables from the relevant year's regulation to construct a measure of price-standardized acute hospital spending. The wage index tables were used to price-standardize outpatient spending. The price-standardization process eliminates the cost variations resulting from geographic payment differences (for inpatient and outpatient claims) and payments for indirect medical education or disproportionate share hospitals (for inpatient claims).

Medicare Physician Fee Schedule (MPFS): The CMS sets the physician fee schedule based on Healthcare Common Procedure Coding System (HCPCS) procedures' relative value units (RVU) for work, practice expense, and malpractice, geographic practice cost indices (GPCI) for each RVU component, conversion factor, and payment modifiers (if applicable). The files used in calculating the MPFS amounts are published on the CMS website quarterly. We used the national RVU weight for each HCPCS code or HCPCS-modifier combination from the relevant year's regulation to construct price-standardized physician service spending. The price-standardization process eliminates the geographic price variations resulting from GPCIs and other geographic-specific payment modifiers.

Geographic crosswalks

Uniform Data System (UDS) Mapper (ZIP code to ZCTA Crosswalk): We used the UDS Mapper jointly developed by the Health Resources and Services Administration, John Snow, Inc., and the American Academy of Family Physicians to convert ZIP codes to ZCTAs since ZIP codes and ZCTAs do not always align. We used this crosswalk file to map ZIP codes of residence provided in MBSF onto ZCTAs used in the ACS data.

© 2025 Hou Y et al. *JAMA Network Open*

CMS SSA to FIPS State and County Crosswalk: The CMS periodically provides crosswalk files that convert the SSA code (state and county) to FIPS county code and Census Core-Based Statistical Area (CBSA). Some CMS data files contain the SSA code but do not provide the FIPS state or county code, or vice versa. We used these crosswalk files to convert the SSA state or county code to the FIPS county code to determine the county-level residence of beneficiaries. We used the CBSA to FIPS county crosswalk included in this file to determine the CBSA of providers for price-standardizing acute inpatient and outpatient spending.

Dartmouth Atlas ZIP Code Crosswalks: The Dartmouth Atlas of Health Care uses Medicare and Medicaid data to provide information and analysis about national, regional, and local markets. We used the ZIP code crosswalk file to assign beneficiaries to HSAs and HRRs based on their ZIP code of residence recorded in MBSF and construct other market-level characteristics.

2. Variable Construction

Standardized spending

In this section, we describe the methodology used to standardize Medicare spending for acute inpatient, outpatient, and carrier claims; total acute care standardized spending was then constructed by summing over these three categories of spending after standardization at the beneficiary year level. We further constructed additional standardized spending by point of service, including hospital outpatient department, Evaluation and Management (E&M), and non-admitted emergency department (ED) visits. The service use section below provides details on identifying each type of service.

The overall goal of standardization is to remove part of variations in Medicare spending that reflect the geographic location of the services being provided and other policy considerations. We followed the CMS approach and simplified algorithms used by previous studies.[1,2]

Inpatient standardized spending: Inpatient spending standardization was based on the Medicare IPPS at the DRG level. Standardized IPPS spending was calculated as a base rate (not wage adjusted) * DRG weight + standardized outlier payments. The base rate, including both operating and capital costs, was obtained from each year's federal regulation.[3] The DRG weight for each diagnosis was updated annually in each year's

© 2025 Hou Y et al. *JAMA Network Open*

regulation. Standardized outlier payments were calculated as outlier payments divided by a wage index factor. The wage index factor was calculated as 0.25 + 0.75 * (wage index for a region each year), where the region is defined as the hospital's CBSA. Following the appendix from Finkelstein, Gentzkow, & Williams (2016),[1] we used the state's rural wage index if a hospital was not located in a CBSA and used the median of the state's urban wage indices if the state did not have a rural wage index (MA, NJ, RI, DC, PR) in a given year. Finally, we adjusted the standardized inpatient spending by a scalar (λ) such that the standardized inpatient spending added up to the aggregated actual inpatient spending.

<u>Physician (Carrier) standardized spending:</u> Physician (carrier) spending standardization was based on MPFS at the HCPCS level. The core components that determine the payment amount were RVUs and a conversion factor that translated the workload needed to furnish a service into dollar amounts. Standardized MPFS spending was calculated as [Work RVU + Practice Expense (PE) RVU + Malpractice (MP) RVU] * conversion factor. Work RVU, PE RVU, MP RVU, and conversion factors were obtained from each year's MPFS. For HCPCS codes with an RVU of zero or did not merge to the fee schedule, we followed Finkelstein, Gentzkow, & Williams (2016) and assigned the RVU weight to be the median carrier spending by HCPCS code-modifier-year, divided by a year-specific price conversion factor.[1] Similarly, we applied the adjustment factor (described above) such that standardized physician spending would add up to the aggregated actual spending on physician services.

<u>Outpatient standardized spending (facility portion):</u> The outpatient spending standardization was based on wage-adjusted ambulatory payment classification weights, where only the labor share (60%) was adjusted by the hospital wage index. Standardized outpatient spending was calculated as payments to hospitals or facilities divided by a wage-adjusted conversion factor [0.4 + 0.6*(wage index for a region each year)]. We applied the final adjustment factor (described above) such that standardized outpatient spending would add up to the aggregated actual spending on outpatient facility use.

Service Use Outcomes

© 2025 Hou Y et al. *JAMA Network Open*

In this section, we described the methodology used to construct variables related to acute care service use. We focused on six utilization outcomes: acute inpatient hospitalization, outpatient facility visits, hospital outpatient department visits, physician visits, E&M visits, and non-admitted ED visits.

Acute inpatient hospitalization: We followed the MSSP specification and identified the acute inpatient stays that meet the following criteria: occurred at acute or critical access hospitals, excluded transfer and death discharge status, excluded beneficiaries who died within 30-day discharge, excluded beneficiaries who left against medical advice, excluded cancer hospitals (IPPS exempt), and excluded duplicated stays. The count of acute inpatient hospitalization was aggregated at the beneficiary year level.

Outpatient facility visits: We identified outpatient facility visits using the outpatient facility claims. To avoid overcounting the total number of visits, we aggregated multiple outpatient claims billed by the same provider on the same day into 1 visit within a beneficiary. The count of outpatient facility visits was aggregated at the beneficiary year level.

Hospital outpatient department visits: We identified hospital outpatient visits using the combination of claim facility type (1= Hospital) and the service classification type code (3= Outpatient, conditional on facility type being 1) using the outpatient facility claims. To avoid overcounting the total number of visits, we aggregated multiple hospital outpatient department claims billed by the same provider on the same day into 1 visit within a beneficiary. The count of hospital outpatient department visits was aggregated at the beneficiary year level.

Physician visits: We identified physician visits using the carrier claims. To avoid overcounting the total number of visits, we aggregated multiple carrier claims billed by the same performing provider on the same day into 1 visit within a beneficiary. The count of physician visits was aggregated at the beneficiary year level.

E&M visits: We identified eligible E&M services in outpatient settings using HCPCS codes 99201-99215, 99241-99245, G0402, G0438, G0439, and G0463 from carrier line level files. We followed the previous study and excluded E&M services that occurred in nursing facilities (HCPCS codes 99304-99318, 99324-99340, and 99341-99350) because nursing facilities are underrepresented in ACO contracts.[4,5] We appended additional E&M services provided in federally qualified health centers, rural health clinics, method 2 critical access

© 2025 Hou Y et al. *JAMA Network Open*

hospitals, and electing teaching amendment hospitals using the combination of CMS Certification Number (CCN), HCPCS code, and type of billing code (combination of claim facility type and the service classification type code for an institutional claim) as specified in the MSSP assignment methodology (version 3) from the outpatient base and revenue center level files.

Non-admitted ED visits: we identified non-admitted ED visits (i.e., ED visits that do not lead to inpatient stays) using revenue center codes of 0450, 0451, 0452, 0456, 0459, or 0981 from outpatient revenue center files and HCPCS codes of 99281-99285 with a place of service code of 23 (Emergency Room - Hospital) from carrier line level files.

Covariates

Chronic Conditions Data Warehouse (CCW) conditions: 27 CCW chronic conditions (end-of-year indicators) include acute myocardial infarction, Alzheimer's disease, Alzheimer's disease and related disorders or senile dementia, anemia, asthma, atrial fibrillation, breast cancer, cataracts, chronic kidney disease, chronic obstructive pulmonary disease, colorectal cancer, depression, diabetes, endometrial cancer, glaucoma, heart failure, hip or pelvic fracture, hyperlipidemia, hyperplasia, hypertension, hypothyroidism, ischemic heart disease, lung cancer, osteoporosis, prostate cancer, rheumatoid arthritis or osteoarthritis, and stroke or transient ischemic attack. We follow the existing literature to group the original 27 CCW conditions into categories (see eTable1).[6] Two eye conditions (cataracts and glaucoma) were excluded from the analyses.

3. Empirical Approach

In this paper, we leveraged variation in exposure to local ACO penetration rates triggered by non-attributed beneficiaries who moved between healthcare markets, defined by hospital service areas (HSAs), to gauge the magnitude of ACO spillovers. This approach draws on two plausibly random sources of variation—both the direction and timing of the move—to separate the impact of ACO penetration from other unobserved local market characteristics.

Main Specification

We used the following linear model to estimate spending or service use changes after moving to areas

with more Medicare beneficiaries in ACOs for non-attributed beneficiaries; the association of ACO penetration with each outcome was then calculated after standardization to a one-standard-deviation change in ACO penetration triggered by beneficiaries moving across markets.

$$Y_{it} = \alpha_i + \lambda_t + \beta \Delta ACOPenetration_i * I_{r(i,t)>0} + \gamma X_{it} + \rho_{r(i,t)} + \varepsilon_{it} \quad (1)$$

The subscripts i, t denote beneficiary and calendar year. The outcome Y_{it} is the log of outcome (spending or service utilization) for beneficiary i in calendar year t. $\Delta ACOPenetration_i$ denotes the change in the beneficiary's exposure to ACO penetration post- compared to the pre-move (after 2012), with the year of moving excluded. $I_{r(i,t)>0}$ is an indicator of being in the post-move period, and the subscript $r(i,t) = t - t_i^*$ indicates the year t relative to the time period when beneficiary i moved (t_i^*). X_{it} is a vector of time-varying beneficiary- and market-level characteristics. α_i, λ_t, $\rho_{r(i,t)}$ are fixed effects for beneficiary, calendar year, and year relative to move, respectively. The overall coefficient of interest is β on the interaction term, $\Delta ACOPenetration_i * I_{r(i,t)>0}$, that captures the estimated changes in outcomes associated with changes in ACO penetration triggered by moving.

Test for Pre-trends

Interpreting $\hat{\beta}$ as a causal estimate of ACO spillover effects relies on two assumptions. First, beneficiaries cannot strategically choose the timing and destination of their move in response to changes in a market's ACO penetration (patient sorting). Although we cannot completely rule out this possibility, it is unlikely for beneficiaries to move in a particular direction according to the ACO penetration level because ACO market penetration was not easily observed. A more plausible channel for sorting is through health problems and access to services. The first assumption allows beneficiaries to move due to a shock to their health status, which is captured by the year relative to move fixed effects; they cannot, however, systematically move from a low to high ACO penetrated market or vice versa because of this health shock. We empirically assessed this assumption by examining whether the change in ACO penetration was correlated with the outcome of interests prior to the move. In equation (2), we substituted the post-move indicators in the interaction term with the year relative to move indicators (up to four years pre-move and two years post-move) and tested whether the

© 2025 Hou Y et al. *JAMA Network Open*

estimated coefficients δ in pre-move years were equal and not statistically significant from zero using a joint *F*-test. Our null hypothesis is $\delta_{-4} = \delta_{-3} = \delta_{-2} = 0$. A significant test result (*P*<0.05) indicates evidence of pre-trend.

$$Y_{it} = \alpha_i + \lambda_t + \delta \Delta ACOPenetration_i * \rho_{r(i,t)} + \gamma X_{it} + \rho_{r(i,t)} + \varepsilon_{it} \quad (2)$$

Detrending Adjustment

We observed downward pre-trends in spending on and use of outpatient facilities, which may indicate patient preferences in choosing the destination of their move (eTable 3). To minimize the impact of pre-trends, we follow the existing literature to detrend the outcome variables.[6–8] Specifically, we estimated a linear pre-trend of change in ACO penetration during the pre-move period:

$$Y_{it} = \alpha_i + \lambda_t + \gamma \Delta ACOPenetration_i * Timeline_t + \rho_{r(i,t)} + \varepsilon_{it} \quad (3)$$

We residualized the outcome variables for all periods by removing the estimated pre-trend (equation 4). $\widehat{Y}_{it}$ was then used as the outcome for the main specification.

$$\widehat{Y}_{it} = \mathrm{Y_{it}} - \hat{\gamma} \Delta ACOPenetration_i * Timeline_t \quad (4)$$

Unobserved Origin-Destination HSA Differences

The second assumption is that no other omitted variables correlate with the change in ACO penetration and spending or service use, conditional on a rich set of patient- and market-level characteristics. We included patient-level fixed effects to account for unobserved patient care-seeking preferences and habits, but the remaining omitted variables may still come from the supply side. The unobserved market differences between origin and destination HSAs, such as provider practice styles and norms, are likely correlated with the change in ACO penetration and, therefore, could bias the estimated ACO spillovers. To assess the extent of this bias, we replaced the patient-level fixed effects with origin-by-destination HSA combination fixed effects $HSA_{(d_i,o_i)}$ to directly account for time-invariant unobservable market differences (equation 5). Identification in this specification comes from observing movers with the exact same origin and destination HSAs but different timing of the move, which results in the variation in $\Delta ACOPenetration_i$. Since the fixed effect was at the HSA

© 2025 Hou Y et al. *JAMA Network Open*

combination level instead of the beneficiary level, we additionally adjusted for the beneficiary's sex, race and ethnicity, dually eligible for Medicaid, disability status, and any end-stage renal disease.

$$Y_{it} = HSA_{(d_i,o_i)} + \lambda_t + \xi \Delta ACOPenetration_i * I_{r(i,t)>0} + \gamma X_{it} + \rho_{r(i,t)} + \varepsilon_{it} \quad (5)$$

eTable 1. Chronic Condition Categories

Chronic Condition Categories	Original CCW Chronic Conditions
Cardiac conditions	Acute myocardial infarction
	Atrial fibrillation
	Heart failure
	Ischemic heart disease
	Hypertension
Alzheimer's disease and related dementias	Alzheimer's disease
	Alzheimer's disease and related disorders or senile dementia
Kidney Disease	Chronic kidney disease
Respiratory conditions	Chronic obstructive pulmonary disease
	Asthma
Endocrine	Diabetes
	Hyperlipidemia
	Anemia
	Hypothyroidism
	Hyperplasia
Hip fracture	Hip or pelvic fracture
Rheumatoid conditions	Osteoporosis
	Rheumatoid arthritis or osteoarthritis
Stroke	Stroke or transient ischemic attack
Cancer	Breast cancer
	Colorectal cancer
	Prostate cancer
	Lung cancer
	Endometrial cancer
Depression	Depression

Abbreviations: CCW, Chronic Conditions Data Warehouse.

eTable 2. Sensitivity Analysis: Estimated Changes After Moving to HSAs With More Medicare Beneficiaries in Accountable Care Organizations for Non-attributed Beneficiaries Using Alternative Specifications (Adjusting for Hierarchical Condition Categories)

	Adjusting for HCC			Without adjusting for HCC		
Outcomes	Estimated change	(95% CI)	Pre-trend (*P* value)	Estimated change	(95% CI)	Pre-trend (*P* value)
Standardized spending (log)						
Acute inpatient	-1.3%	(-3.1% to 0.5%)	0.99	-1.6%	(-3.5% to 0.3%)	0.83
Outpatient facility	-4.4%	(-5.9% to -2.8%)	0.36	-5.0%	(-6.6% to -3.3%)	0.27
Hospital outpatient department	-5.1%	(-6.9% to -3.3%)	0.53	-5.4%	(-7.2% to -3.6%)	0.39
Physician service	1.5%	(0.9% to 2.1%)	0.35	1.6%	(1% to 2.2%)	0.67
Evaluation & Management	0.2%	(-0.2% to 0.6%)	0.22	0.3%	(-0.2% to 0.7%)	0.20
Non-admitted ED	-1.8%	(-3.5% to -0.2%)	0.88	-2.1%	(-3.8% to -0.4%)	0.71
Total acute care	0.8%	(0.3% to 1.4%)	0.79	0.8%	(0.2% to 1.4%)	0.90
Service use (log)						
Acute hospitalization	-0.1%	(-0.2% to 0.1%)	0.99	-0.1%	(-0.3% to 0.1%)	0.81
Outpatient visit	-2.2%	(-2.7% to -1.7%)	0.32	-2.4%	(-2.9% to -1.9%)	0.23
Hospital outpatient department visit	-2.0%	(-2.4% to -1.5%)	0.15	-2.1%	(-2.6% to -1.6%)	0.10
Physician visit	1.0%	(0.6% to 1.4%)	0.51	1.0%	(0.6% to 1.4%)	0.88
Evaluation & Management	0.3%	(-0.04% to 0.6%)	0.51	0.3%	(0.01% to 0.6%)	0.51
Non-admitted ED	-0.3%	(-0.5% to 0%)	0.93	-0.3%	(-0.6% to -0.1%)	0.69

Abbreviations: HSA, Hospital Service Areas; HCC, Hierarchical Condition Category. ED, Emergency Department.

eTable2 reports the estimated spending and service use changes and 95%CIs on the interactions between changes in ACO penetration and an indicator of being in the post-move period. The regression controls for the five-year age bin, time-varying beneficiary and market characteristics, and fixed effects for the beneficiary, relative year to move, and calendar year. Standard errors are clustered at the beneficiary level. Pre-trend tests reported the *P* value from a joint *F* test of the estimated coefficients during the pre-move period after the detrending adjustment. $P<0.05$ indicates evidence of pre-trend.

© 2025 Hou Y et al. *JAMA Network Open*

eTable 3. Sensitivity Analysis: Estimated Changes After Moving to HSAs With More Medicare Beneficiaries in Accountable Care Organizations for Non-attributed Beneficiaries Using Alternative Specifications (Without Detrending)

	Without detrending			With detrending		
Outcomes	Estimated change	(95% CI)	Pre-trend (*P* value)	Estimated change	(95% CI)	Pre-trend (*P* value)
Standardized spending (log)						
Acute inpatient	0.9%	(-0.9% to 2.7%)	0.53	-1.6%	(-3.5% to 0.3%)	0.83
Outpatient facility	-6.6%	(-8.2% to -5%)	0.01	-5.0%	(-6.6% to -3.3%)	0.27
Hospital outpatient department	-6.3%	(-8.2% to -4.5%)	0.15	-5.4%	(-7.2% to -3.6%)	0.39
Physician service	0.8%	(0.3% to 1.4%)	0.76	1.6%	(1% to 2.2%)	0.67
Evaluation & Management	-0.6%	(-1.1% to -0.2%)	0.00	0.3%	(-0.2% to 0.7%)	0.20
Non-admitted ED	-1.8%	(-3.5% to -0.2%)	0.88	-2.1%	(-3.8% to -0.4%)	0.71
Total acute care	-0.1%	(-0.7% to 0.4%)	0.49	0.8%	(0.2% to 1.4%)	0.90
Service use (log)						
Acute hospitalization	0.03%	(-0.1% to 0.2%)	0.86	-0.1%	(-0.3% to 0.1%)	0.81
Outpatient visit	-3.3%	(-3.8% to -2.8%)	0.00	-2.4%	(-2.9% to -1.9%)	0.23
Hospital outpatient department visit	-2.4%	(-2.9% to -1.9%)	0.01	-2.1%	(-2.6% to -1.6%)	0.10
Physician visit	0.7%	(0.3% to 1%)	0.82	1.0%	(0.6% to 1.4%)	0.88
Evaluation & Management	-0.2%	(-0.5% to 0.1%)	0.03	0.3%	(0.01% to 0.6%)	0.51
Non-admitted ED	-0.3%	(-0.5% to 0%)	0.91	-0.3%	(-0.6% to -0.1%)	0.69

Abbreviations: HSA, Hospital Service Areas; ED, Emergency Department.

eTable3 reports the estimated spending and service use changes and 95%CIs on the interactions between changes in ACO penetration and an indicator of being in the post-move period. The regression controls for the five-year age bin, time-varying beneficiary and market characteristics, and fixed effects for the beneficiary, relative year to move, and calendar year. Standard errors are clustered at the beneficiary level. Pre-trend tests reported the *P* value from a joint *F* test of the estimated coefficients during the pre-move period with and without the detrending adjustment. $P<0.05$ indicates evidence of pre-trend.

© 2025 Hou Y et al. *JAMA Network Open*

eTable 4. Sensitivity Analysis: Estimated Changes After Moving to HSAs With More Medicare Beneficiaries in Accountable Care Organizations for Non-attributed Beneficiaries Using Alternative Specifications (Outcomes without Log Transformation)

	Outcomes without log transformation		
Outcomes	Estimated change	(95% CI)	Pre-trend (*P* value)
Standardized spending, $			
Acute inpatient	8	(-26 to 42)	0.43
Outpatient facility	-32	(-50 to -14)	0.04
Hospital outpatient department	-14	(-28 to -1)	0.43
Physician service	41	(21 to 62)	0.71
Evaluation & Management	-4	(-7 to -1)	0.07
Non-admitted ED	-3	(-7 to 1)	0.47
Total acute care	33	(-23 to 89)	0.95
Service use (no.)			
Acute hospitalization	0.0004	(-0.002 to 0.003)	0.88
Outpatient visit	-0.19	(-0.23 to -0.16)	0.00
Hospital outpatient department visit	-0.10	(-0.12 to -0.07)	0.02
Physician visit	0.24	(0.15 to 0.32)	0.32
Evaluation & Management	-0.01	(-0.04 to 0.02)	0.18
Non-admitted ED	-0.01	(-0.01 to -0.001)	0.90

Abbreviations: HSA, Hospital Service Areas; ED, Emergency Department.

eTable4 reports the estimated spending and service use changes and 95%CIs on the interactions between changes in ACO penetration and an indicator of being in the post-move period. The regression controls for the five-year age bin, time-varying beneficiary and market characteristics, and fixed effects for the beneficiary, relative year to move, and calendar year. Standard errors are clustered at the beneficiary level. Pre-trend tests reported the *P* value from a joint *F* test of the estimated coefficients during the pre-move period without the detrending adjustment.

© 2025 Hou Y et al. *JAMA Network Open*

eFigure 1. Diagram of Study Sample Selection

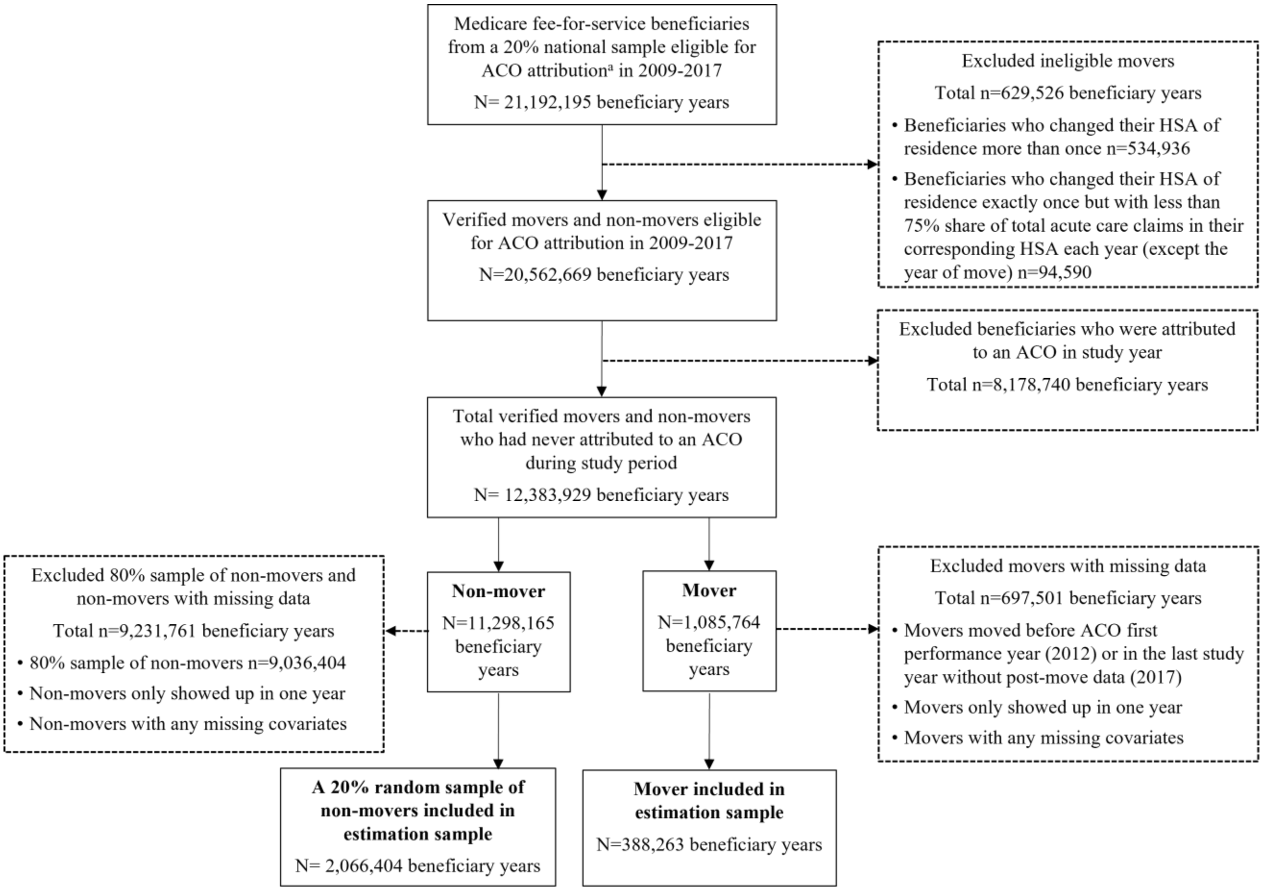

Abbreviations: ACO, Accountable Care Organizations; HSA, Hospital Service Areas.

Eligibility for ACO attribution includes beneficiaries with continuous enrollment in Medicare Parts A and B, not enrolled in Medicare Advantage (MA), and who had at least one eligible Evaluation & Management (E&M) service provided by primary care physicians each year.

© 2025 Hou Y et al. *JAMA Network Open*

eFigure 2. Share of Claims Billed in Destination Areas by Year Relative to Move

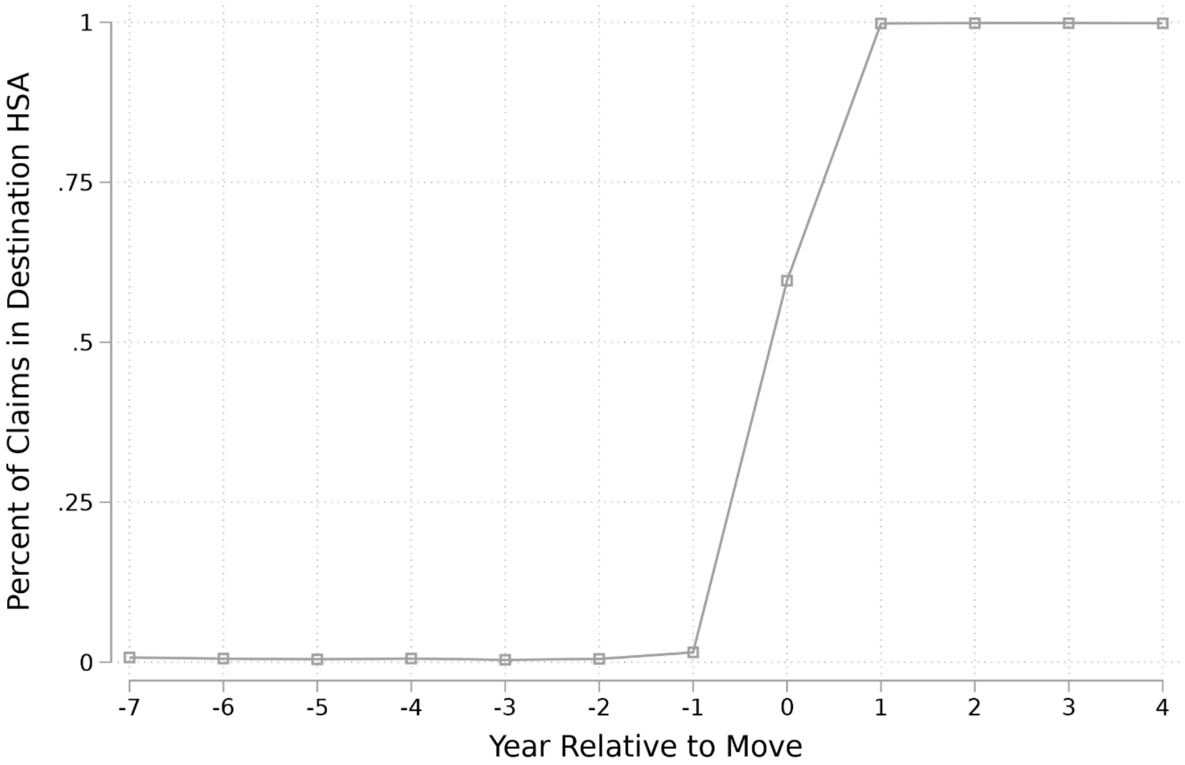

Abbreviations: HSA, Hospital Service Area.

eFigure2 plots the share of acute care claims (inpatient, outpatient, and carrier) billed in destination HSA by year relative to move among movers. Movers were excluded if they had less than 75% of their total claims billed in their corresponding HSA of residence recorded in the Master Beneficiary Summary File each year except the year of the move.

© 2025 Hou Y et al. *JAMA Network Open*

eFigure 3. Distribution of Changes in the HSA-level ACO Penetration Induced by Movers

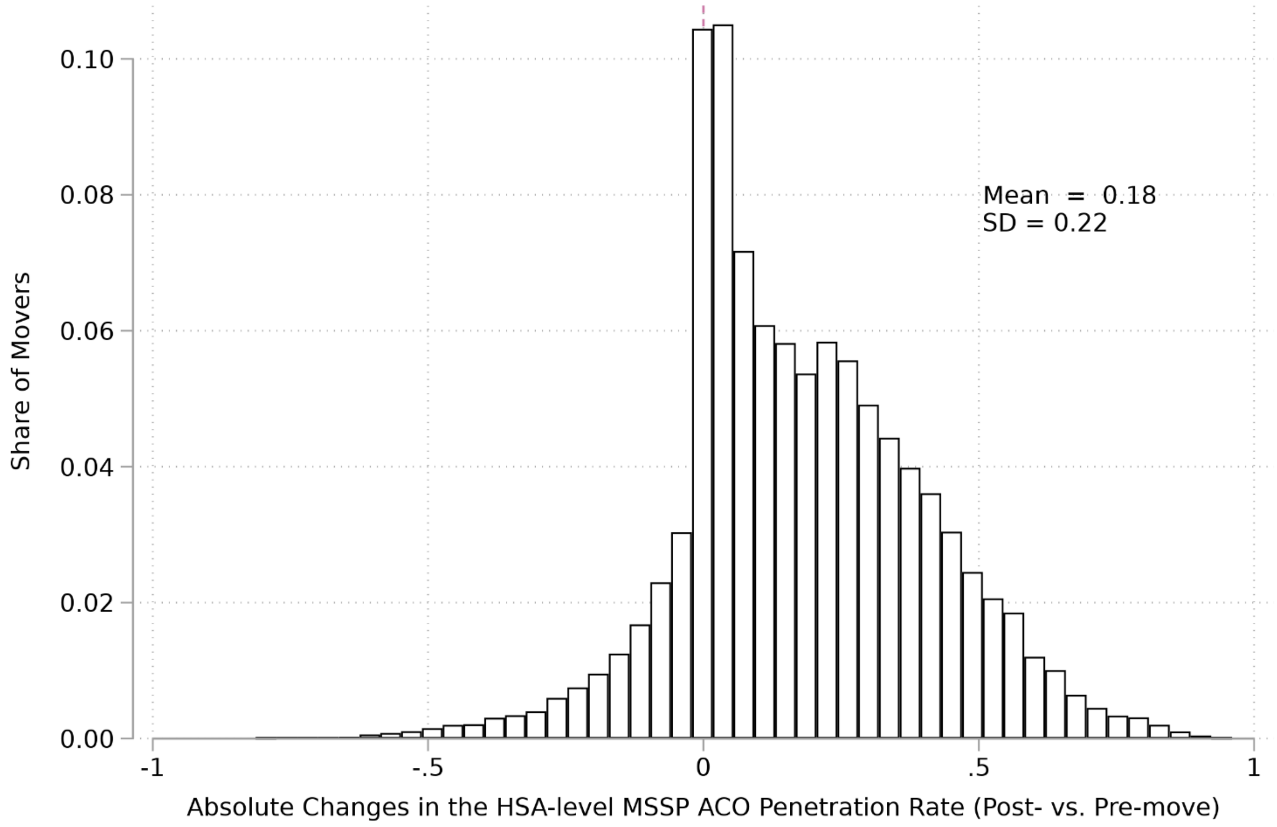

Abbreviations: MSSP, Medicare Shared Savings Program; ACO, Accountable Care Organization; HSA, Hospital Service Area.

The absolute percentage point changes in the HSA-level ACO penetration rate in the post-move compared to the pre-move period among non-attributed beneficiaries. Beneficiaries can move from low- to high-ACO penetrated areas (positive values), or vice versa (negative values). HSA-level ACO penetration is defined as the share of eligible beneficiaries derived from a 20% Medicare sample assigned to an MSSP ACO within an HSA year.

© 2025 Hou Y et al. *JAMA Network Open*

eFigure 4. Variation in Accountable Care Organization Penetration at the HSA and HRR Levels

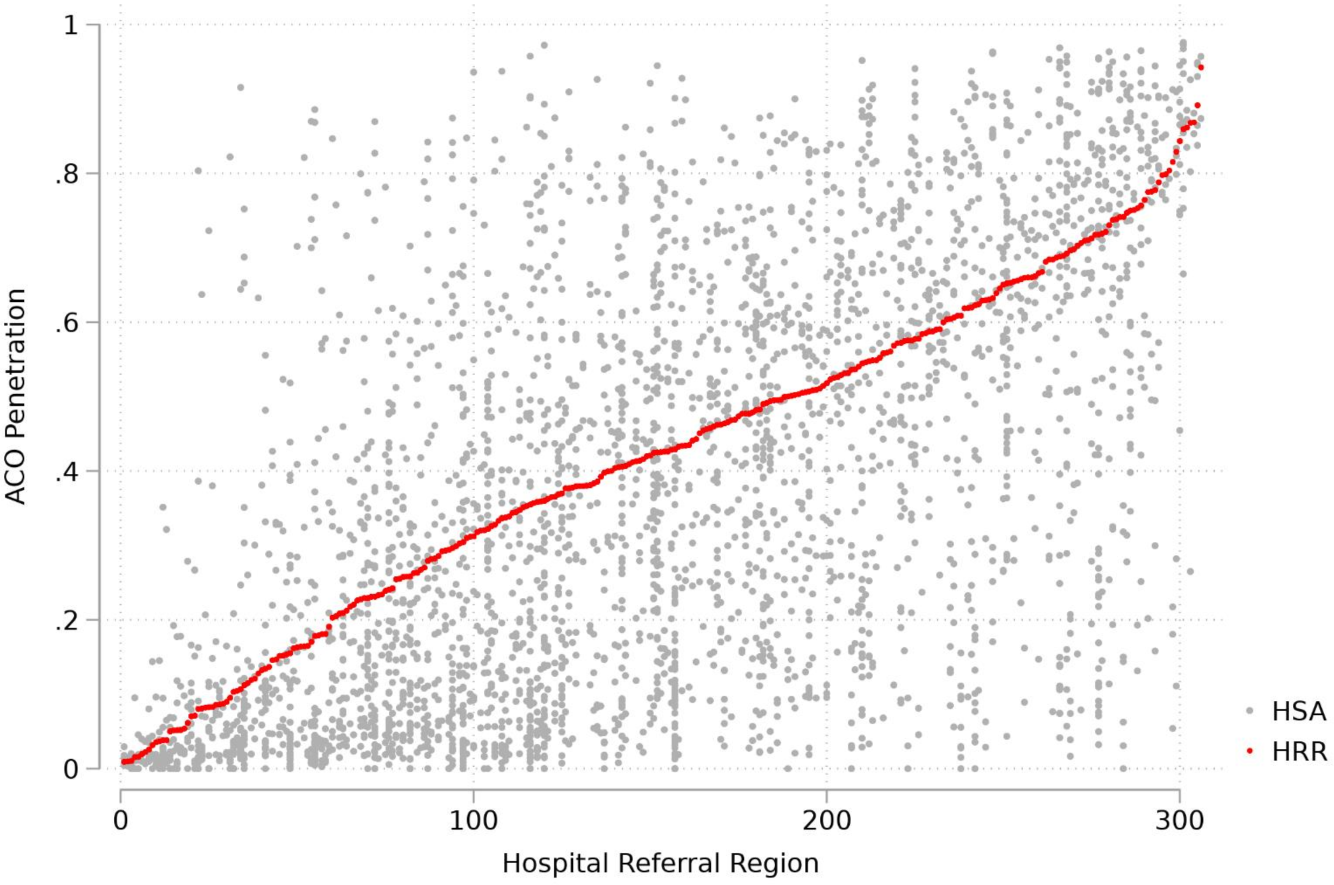

Abbreviations: ACO, Accountable Care Organizations; HSA, Hospital Service Areas; HRR, Hospital Referral Region.

Scatterplot of variation in ACO penetration rates at the HRR and HSA levels in 2017. Each red dot represents ACO penetration at the HRR level, while each gray dot represents the ACO penetration at the HSA level. The x-axis represents 306 HRR sorted by ACO penetration.

© 2025 Hou Y et al. *JAMA Network Open*

eFigure 5. Sensitivity Analysis: Estimated Spending Changes After Moving to HRRs With More Medicare Beneficiaries in Accountable Care Organizations for Non-attributed Beneficiaries

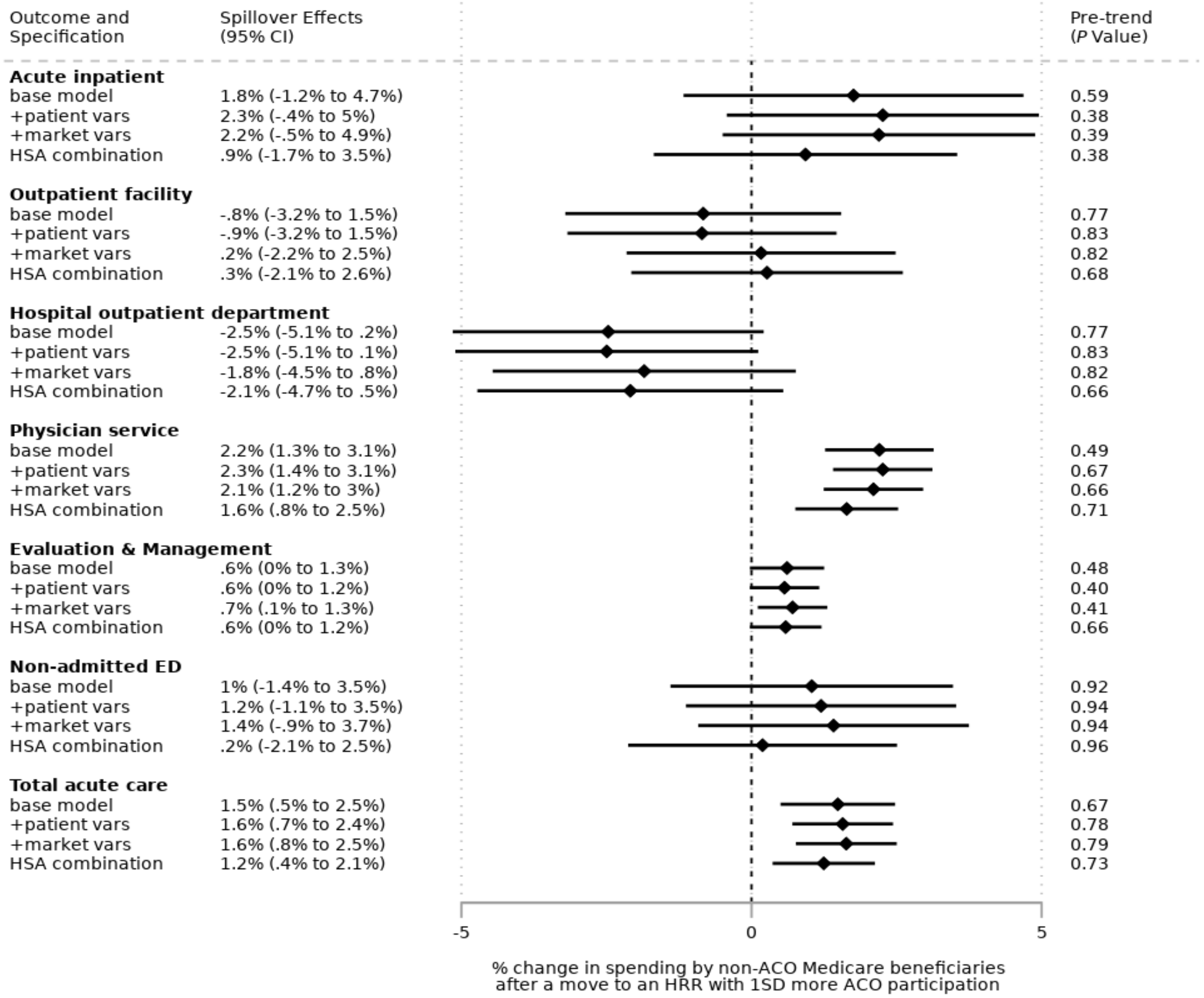

Abbreviations: HRR, Hospital Referral Region. ED, Emergency Department.

Estimated spending changes after moving to HRRs with more Medicare beneficiaries in ACO for non-attributed beneficiaries, by different specifications. All regressions controlled for age (five-year age bin), beneficiary (specification 1-3), or HRR combination (specification 4) fixed effects, relative year fixed effects, and calendar year fixed effects. Pre-trend tests reported the *P* value from a joint *F* test of the estimated coefficients during the pre-move period after the detrending adjustment. *P*<0.05 indicates evidence of pre-trend.

© 2025 Hou Y et al. *JAMA Network Open*

eFigure 6. Sensitivity Analysis: Estimated Service Use Changes After Moving to HRRs With More Medicare Beneficiaries in Accountable Care Organizations for Non-attributed Beneficiaries

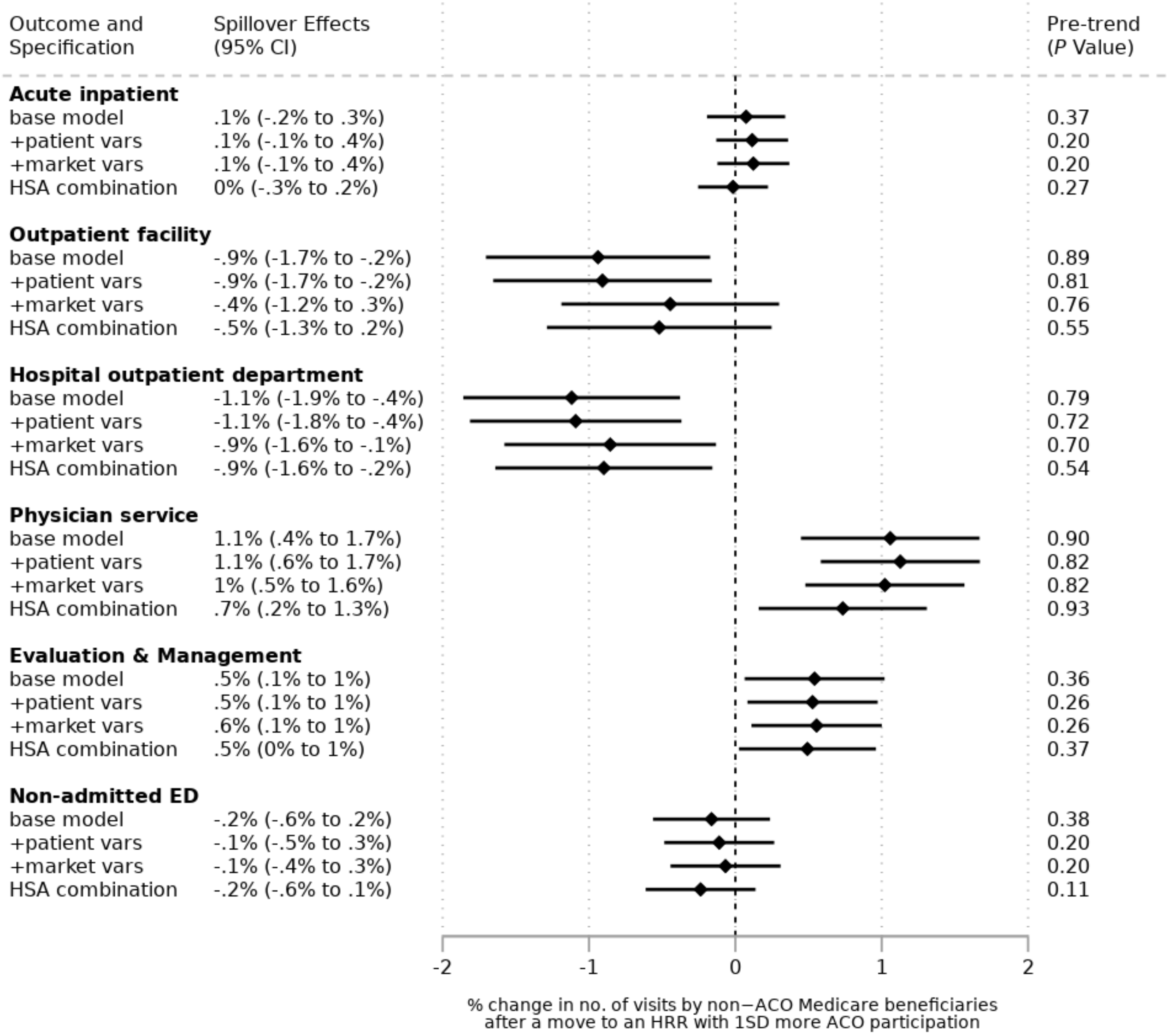

Abbreviations: HRR, Hospital Referral Region. ED, Emergency Department.

Estimated service use changes after moving to HRRs with more Medicare beneficiaries in ACO for non-attributed beneficiaries, by different specifications. All regressions controlled for age (five-year age bin), beneficiary (specification 1-3), or HRR combination (specification 4) fixed effects, relative year fixed effects, and calendar year fixed effects. Pre-trend tests reported the *P* value from a joint *F* test of the estimated coefficients during the pre-move period after the detrending adjustment. $P<0.05$ indicates evidence of pre-trend.

© 2025 Hou Y et al. *JAMA Network Open*

eFigure 7. Event Study Graph: Estimated Spending Changes After Moving to Hospital Service Areas [HSA] With More Medicare Beneficiaries in Accountable Care Organizations [ACO] for Non-attributed Beneficiaries

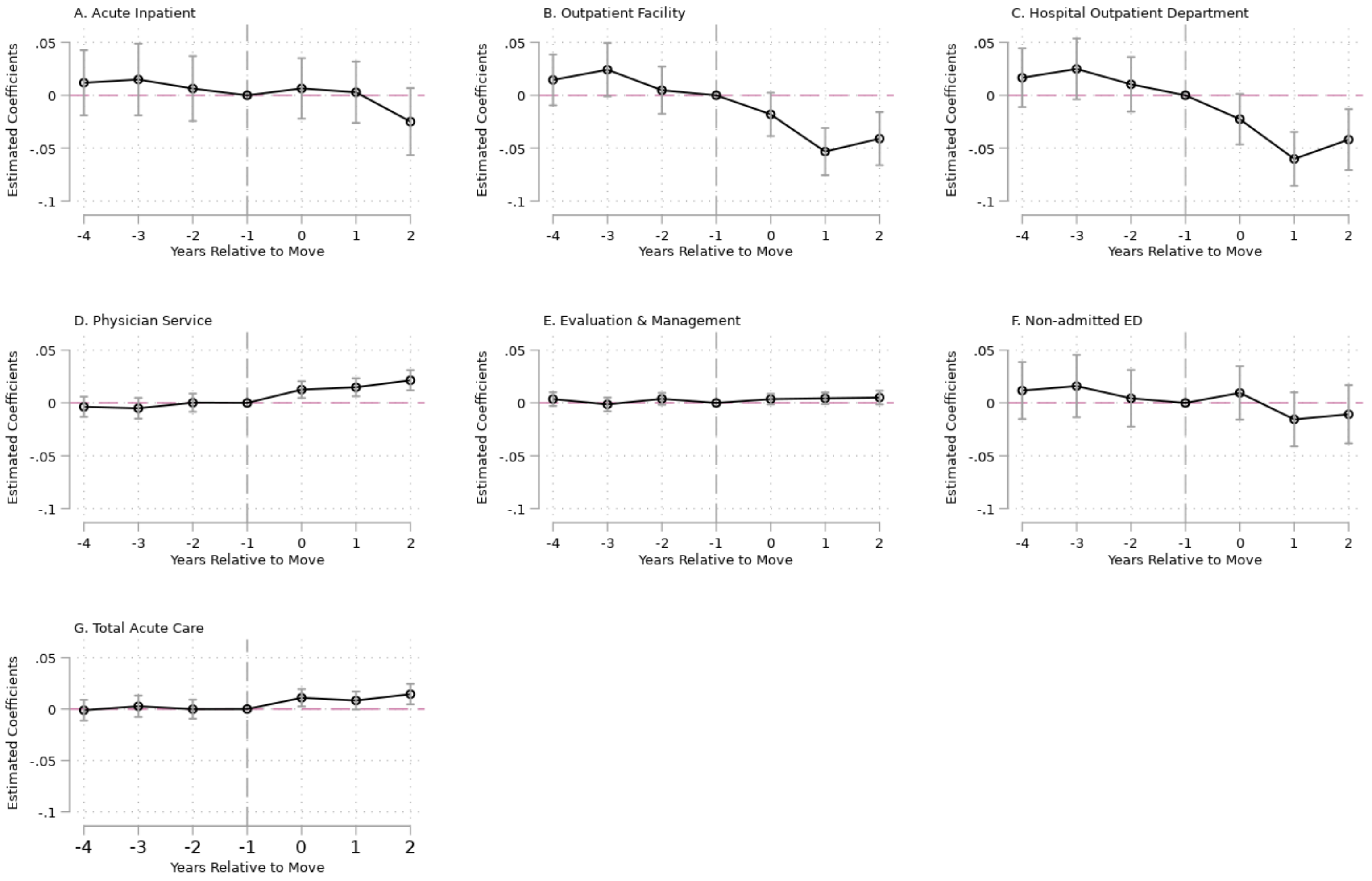

eFigure 7 plots the estimated spending changes and 95%CIs on the interactions between changes in ACO penetration and a series of indicators for 4 years before and 2 years after the move. Endpoints are binned up at 4 years pre-move and 2 years post-move. The estimated coefficient on year -1 (excluded) is normalized to 0. The regression controls for the five-year age bin, time-varying beneficiary and market characteristics, and fixed effects for the beneficiary, relative year to move, and calendar year. Standard errors are clustered at the beneficiary level.

© 2025 Hou Y et al. *JAMA Network Open*

eFigure 8. Event Study Graph: Estimated Service Use Changes After Moving to HSAs With More Medicare Beneficiaries in Accountable Care Organizations for Non-attributed Beneficiaries

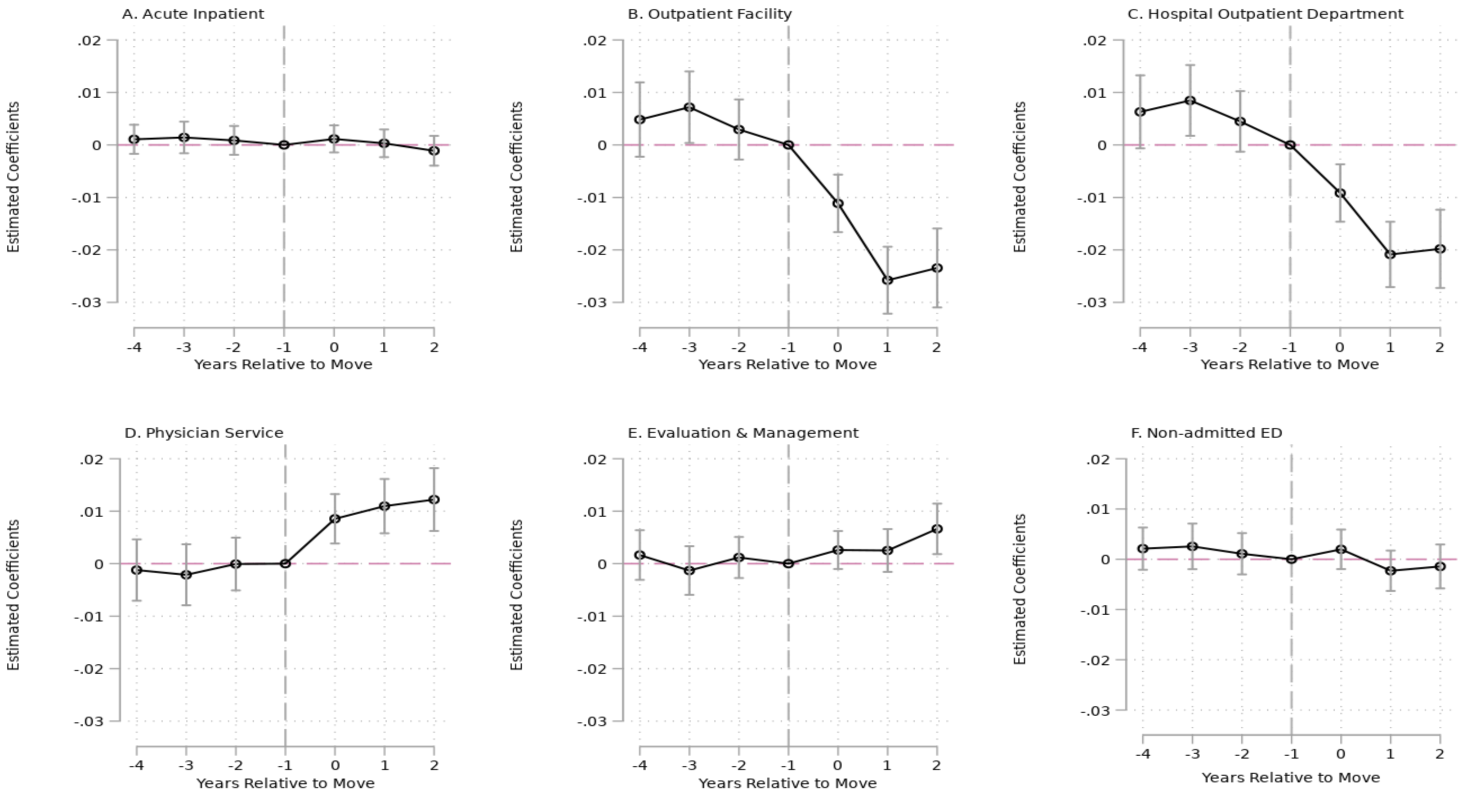

eFigure 8 plots the estimated changes and 95%CIs on the interactions between changes in ACO penetration and a series of indicators for 4 years before and 2 years after the move. Endpoints are binned up at 4 years pre-move and 2 years post-move. The estimated coefficient on year -1 (excluded) is normalized to 0. The regression controls for the five-year age bin, time-varying beneficiary and market characteristics, and fixed effects for the beneficiary, relative year to move, and calendar year. Standard errors are clustered at the beneficiary level.

© 2025 Hou Y et al. *JAMA Network Open*

eReferences

1. Finkelstein A, Gentzkow M, Williams H. Sources of Geographic Variation in Health Care: Evidence From PatientMigration*. *The Quarterly Journal of Economics*. 2016;131(4):1681-1726. doi:10.1093/qje/qjw023

2. Gottlieb DJ, Zhou W, Song Y, Andrews KG, Skinner JS, Sutherland JM. Prices Don't Drive Regional Medicare Spending Variations. *Health Affairs*. 2010;29(3):537-543. doi:10.1377/hlthaff.2009.0609

3. Acute Inpatient - Files for Download | CMS. Accessed November 22, 2024. https://www.cms.gov/medicare/payment/prospective-payment-systems/acute-inpatient-pps/acute-inpatient-files-download

4. McWilliams JM, Hatfield LA, Chernew ME, Landon BE, Schwartz AL. Early performance of accountable care organizations in medicare. *The New England Journal of Medicine*. 2016;374(24):2357-2366. doi:10.1056/NEJMsa1600142

5. McWilliams JM, Hatfield LA, Landon BE, Hamed P, Chernew ME. Medicare Spending after 3 Years of the Medicare Shared Savings Program. *The New England Journal of Medicine*. 2018;379(12):1139-1149. doi:10.1056/NEJMsa1803388

6. McConnell KJ, Edelstein S, Hall J, et al. Access, Utilization, and Quality of Behavioral Health Integration in Medicaid Managed Care. *JAMA Health Forum*. 2023;4(12):e234593. doi:10.1001/jamahealthforum.2023.4593

7. Goodman-Bacon A. The Long-Run Effects of Childhood Insurance Coverage: Medicaid Implementation, Adult Health, and Labor Market Outcomes. *American Economic Review*. 2021;111(8):2550-2593. doi:10.1257/aer.20171671

8. Agha L, Zeltzer D. Drug Diffusion through Peer Networks: The Influence of Industry Payments. *American Economic Journal: Economic Policy*. 2022;14(2):1-33. doi:10.1257/pol.20200044

© 2025 Hou Y et al. *JAMA Network Open*
