# Supplementary material for: Spending Changes After Moving to Areas With Greater ACO Participation Among Nonattributed Medicare Beneficiaries
Source: JAMA Netw Open. 2025 Feb 20;8(2):e2458311. doi: 10.1001/jamanetworkopen.2024.58311 (PMC11843370; doi:10.1001/jamanetworkopen.2024.58311)
Supplement: Supplement 2. — Data Sharing Statement [file jamanetwopen-e2458311-s002.pdf]

Data Sharing Statement

Hou. Spending Changes After Moving to Areas With Greater Accountable Care Organization Participation Among Nonattributed Medicare Beneficiaries. *JAMA Netw Open*. Published February 20, 2025. doi:10.1001/jamanetworkopen.2024.58311

Data
Data available: No

Additional Information
Explanation for why data not available: The data used for the study is a 20% Medicare claims data and cannot be made public at the patient-level due to data use agreements with Centers for Medicare and Medicaid Services.
